# Computational Analysis of a Wind Turbine Blade for Different Advanced Materials

**DOI:** 10.3390/ma18112447

**Published:** 2025-05-23

**Authors:** Panagiotis F. Fragkos, Efstathios E. Theotokoglou

**Affiliations:** Laboratory of Testing and Materials, Department of Mechanics, School of Applied Mathematical and Physical Sciences, National Technical University of Athens, Zografou Campus, 15773 Athens, Greece; fragkospanos@mail.ntua.gr

**Keywords:** computational analysis, advanced materials, fiber-reinforced composites, IEA 15 MW wind turbine, wind turbine blades, fluid–structure interaction, offshore structures

## Abstract

As wind turbine rotors grow in size and Greece advances its offshore wind energy initiatives, this study analyzes the structural behavior of offshore wind turbine blades using fluid–structure interaction (FSI) methods. The blade skin and shear webs of the International Energy Agency (IEA) 15 MW wind turbine, assumed to operate in the Aegean Sea, are examined. Computational fluid dynamics (CFD) simulations are conducted for two steady-state wind speeds based on local weather data, followed by finite element analysis (FEA) to assess advanced materials in terms of strength, cost, and carbon footprint. This is the first study to evaluate bamboo- and basalt-based composite materials under Greek offshore wind conditions using FSI methods. Bamboo composites are affordable and sustainable, but their limited durability reduces their viability in offshore environments. The simulation results indicate that using bamboo composites as blade skin may lead to damage due to the excessive loads on offshore wind turbine blades. In contrast, basalt fiber composites are also environmentally viable and offer superior strength, corrosion resistance, and long-term performance, making them a promising alternative. However, their naturally high density may impact the overall weight of the structure. This study concludes that offshore wind technology in the Aegean is feasible but remains costly and environmentally demanding. The further development and adoption of basalt fibers may serve as a gateway to more environmentally friendly offshore structures.

## 1. Introduction

Wind energy plays a crucial role in Greece’s renewable energy strategy, contributing 23% of the country’s total energy demand in 2024. To accelerate offshore wind deployment, the Greek government has set a target of at least 2 GW of offshore wind capacity by 2030, as outlined in the National Energy and Climate Plan (NECP). Harnessing Greece’s substantial wind resources is essential for sustainable development, energy security, and climate change mitigation [1].

Beyond environmental imperatives, economic and regulatory factors influence Greece’s renewable energy transition. The European Union (EU) and the United Nations (UN) impose climate targets through agreements such as the Paris Agreement [2], aiming for Europe to become the first climate-neutral continent by 2050. Meeting these objectives requires a shift from lignite-based energy production to renewable alternatives.

**Figure 1:**
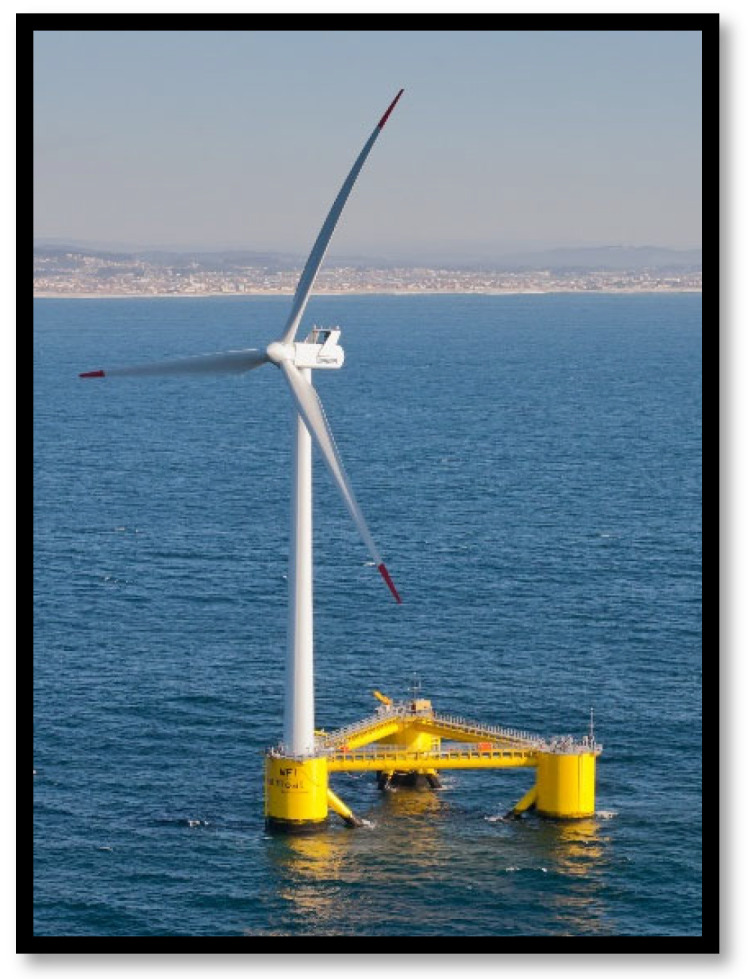
Offshore wind turbine with floating base [5].

Despite ambitious targets, offshore wind energy adoption in Greece faces challenges. The deep waters of the Aegean Sea complicate fixed-foundation turbine installation, while high material costs, complex construction processes, and maintenance requirements further impact feasibility. Additionally, integrating offshore wind farms into the national grid presents logistical and financial constraints [3]. In their research, Ouria et al. [4] concluded that the capacity factor, hub height, and blade diameters are simultaneously the most decisive in the productivity of alternative wind turbines (Figure 1).

An alternative to offshore wind turbines is the deployment of underwater tidal turbines, such as those featured in the NH1 Tidal Project [6]. However, unlike regions such as northern France, the Greek seas generally exhibit lower tidal energy potential, which reduces the efficiency and economic feasibility of tidal technologies in this context. In contrast, offshore wind energy benefits from more favorable wind conditions in Greece, as well as a more mature technological framework, stronger investment interest, and active development both regionally and in the literature.

Notably, wind energy development prioritizes two key design advancements: aerodynamic optimization and the scaling of wind turbines. Improved blade designs enhance aerodynamic efficiency, with a notable innovation being the integration of curved winglets, a feature commonly used in aircraft to reduce drag forces. However, despite their potential to slightly improve energy capture, winglets have not been widely adopted in wind turbines due to limited efficiency gains [7]. Additionally, increasing the rotor diameter enhances energy yield but introduces structural and logistical challenges. Onshore turbines face transportation constraints, particularly in mountainous regions, whereas offshore turbines allow for larger designs with fewer restrictions. While offshore wind turbines can theoretically scale without major constraints, practical challenges persist. Greece’s first floating wind farm is expected to be operational east of Crete by 2028 [8], and large-scale development may introduce unforeseen environmental and engineering complexities, including potential interactions with marine ecosystems.

Wind turbine blades are subject to various forms of damage, particularly in regions experiencing high aerodynamic and mechanical loads. According to Katsaprakakis et al. [9], the most common locations for damage include the leading edge, trailing edge, blade tip, and root section. The leading edge serves as the primary impact zone for airborne particles, rain, and hail, making it highly susceptible to erosion. The trailing edge, where airflow from both blade surfaces converges, is prone to fatigue cracking. The blade tip, being the outermost section, experiences high stress and is frequently struck by lightning. The root section, which connects the blade to the hub, is particularly vulnerable to cyclic loading, stress concentration [10], and adhesive joint failures.

Offshore wind turbines face additional environmental challenges compared to their onshore counterparts [9]. High wind speeds and saline exposure accelerate leading edge erosion, increasing material degradation rates. Corrosion effects, particularly due to saltwater exposure, contribute to structural wear, especially at blade joints. Furthermore, biofouling occurs as marine organisms accumulate on blade surfaces, altering aerodynamics and increasing mechanical loads. These challenges necessitate advanced material selection and targeted maintenance strategies to ensure offshore turbine longevity.

**Figure 2:**
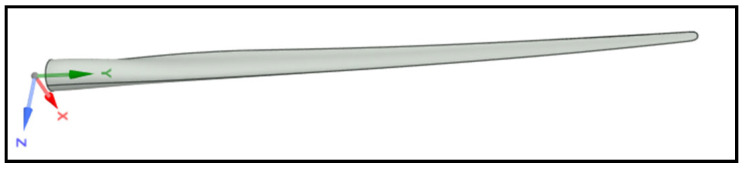
IEA 15 MW wind turbine blade CAD model.

Recent developments in computational engineering have advanced the analysis of offshore wind turbines. The International Energy Agency (IEA) introduced the IEA 15-megawatt (MW) Offshore Reference Wind Turbine (IEA-15-240-RWT) [11], a standardized model for offshore turbine research. Developed in collaboration with the Technical University of Denmark (DTU) and the National Renewable Energy Laboratory (NREL), this model supports industry-research cooperation and innovation in turbine design. The computer-aided design (CAD) model of the blade has been utilized in a wide variety of studies of offshore wind turbine blade behavior (Figure 2). Since its introduction in 2020, large-scale floating wind turbine studies have gained significant traction in both research and industry applications [12,13,14].

This study builds upon methodologies established in previous research, including the work of Theotokoglou and Xenakis [15] and Nebiewa et al. [16]. Their research utilizes a geometric model of a wind turbine blade, incorporating periodic boundary conditions to simulate rotor performance while enabling localized material studies. Both studies use fluid–structural interaction (FSI) methods to determine the displacement and stresses of the blade for exposure to various wind speeds.

The present study focuses on the computational analysis of a theoretical candidate wind turbine blade for floating offshore wind farms. Given the limited availability of detailed internal geometry, emphasis is placed on the outer casing (blade shell or skin) as the primary structural component under investigation. The study examines the aerodynamic loads acting on the blade under nominal operating conditions (“rated conditions”) and extreme conditions (“feathered” operation [17]). Additionally, a comparative assessment of different blade materials is conducted to evaluate their static performance.

Beyond structural analysis, this research also includes a preliminary economic and environmental evaluation of floating wind turbine deployment in Greek waters. This holistic approach aims to provide an initial assessment of the feasibility and performance of floating offshore wind turbines in the Greek seas, supporting future advancements in the country’s offshore wind energy sector.

## 2. Materials and Methods

The cross-section of a standard wind turbine blade incorporates an internal support structure consisting of beams arranged in a box-spar configuration. Two of these beams, oriented perpendicular to the cross-section, are referred to as shear webs. The shear webs are structurally connected by two additional beams, known as spar caps [18].

**Figure 3:**
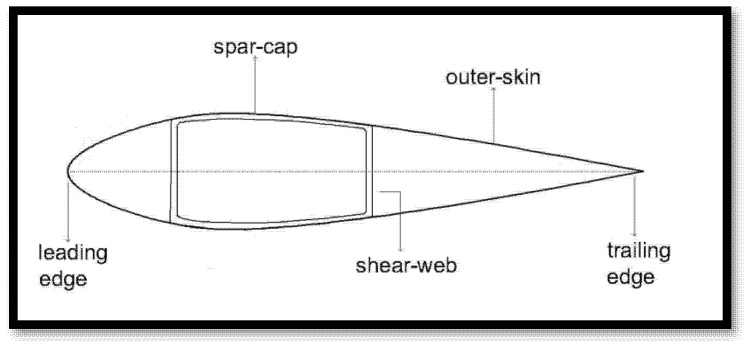
Internal structure of a typical wind turbine blade in section [18].

Shear webs play a crucial role in enhancing the blade’s mechanical performance, enabling it to behave like a solid beam while significantly reducing mass. Without them, the blade would lack the necessary structural integrity to withstand the substantial aerodynamic and gravitational forces imposed by wind loads (Figure 3).

In this study, only the blade skin (casing) and shear webs are modeled. This approach ensures a balance between computational efficiency and accuracy, yielding realistic and reliable results for static analysis [14,18].

**Figure 4:**
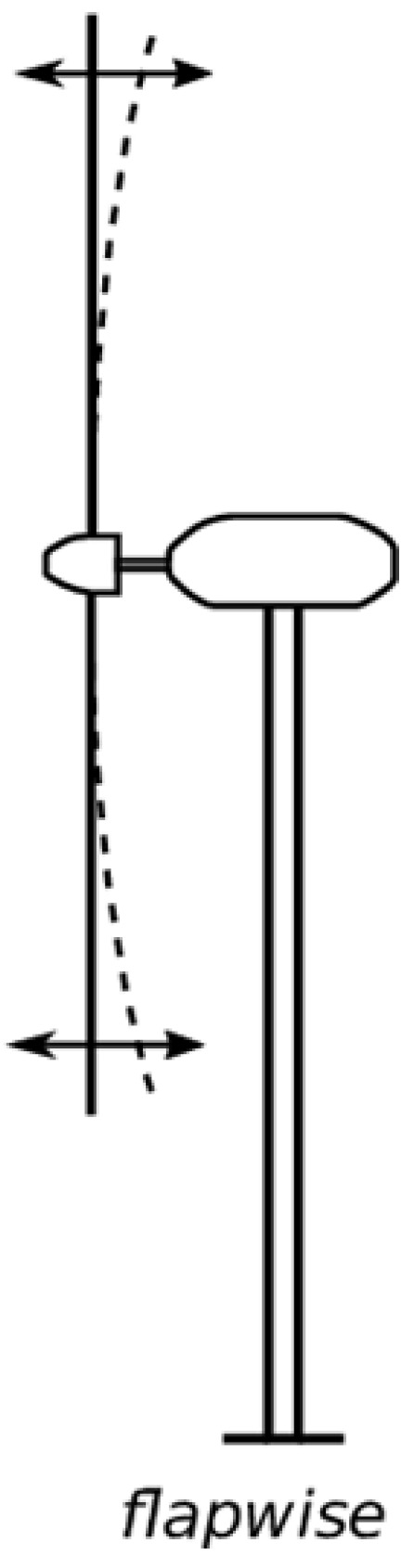
Flapwise bending moment on wind turbine blades [19].

Modern wind turbine blades are predominantly constructed from composite materials, which consist of two or more constituent components that, when combined, exhibit superior mechanical properties not attainable individually. One critical property provided by these materials is resistance to aerodynamic forces, particularly in the flapwise direction (bending forces). The shear webs contribute significantly to this resistance (Figure 4). While resistance to flapwise aerodynamic forces is critical for wind turbine blades, this property is fundamentally governed by the material’s modulus of elasticity *E*. A high modulus of elasticity *E* enhances structural stiffness, limits deflection, and contributes to favorable dynamic behavior by raising the blade’s natural frequency. The latter is desirable because it prevents resonance with operating frequencies.

The selection of suitable materials is a key consideration in wind turbine blade design. As turbine sizes increase, the mass of the blades rises rapidly, making material choice a crucial factor in maintaining structural efficiency. Additionally, excessive bending moments must be avoided to prevent blade-to-tower collisions. Furthermore, offshore wind turbine blades must endure harsh environmental conditions and sustain periodic loads due to continuous rotor rotation over extended operational periods [20].

The composite materials used in wind turbine blades are fiber-reinforced composites. These materials consist of a reinforcement component, which enhances mechanical properties, and a matrix component, which ensures optimal load transfer and structural integrity. Most fiber-reinforced composites used in wind energy applications employ a polymer matrix. The reinforcing fibers are selected for their high stiffness, high fracture toughness, and low density, making them ideal for large-scale wind turbine blades.

Fiberglass is a composite material consisting of glass fibers—either continuous or discontinuous—embedded in a polymeric matrix. This type of composite is widely produced due to its favorable mechanical properties, low cost, and compatibility with various manufacturing techniques for glass-reinforced plastics [15].

Carbon fibers are high-performance reinforcing agents extensively used in advanced polymer matrix composites beyond traditional fiberglass applications. Among all fibrous reinforcement materials, carbon fibers exhibit the highest specific modulus of elasticity and the highest specific strength [15]. They retain exceptional tensile strength and thermal resistance, making them particularly suitable for demanding environments. Additionally, carbon fibers are resistant to moisture [10], solvents, acids, and bases at room temperature, which is a significant advantage for offshore wind turbine applications.

Aramid fibers are known for their high strength and elasticity. They offer superior strength-to-weight ratios compared to metals, making them valuable for lightweight structural applications [15]. A notable drawback of aramid fibers is their susceptibility to moisture absorption [21], which may affect long-term performance in offshore conditions.

Basalt fiber composites are gaining attention as a promising alternative to conventional reinforcement materials in wind turbine blades. Derived from volcanic rock through a high-temperature melting and extrusion process, basalt fibers offer a unique combination of high strength, durability, and resistance to environmental degradation, making them particularly suitable for wind energy applications [22].

Table 1 presents the fundamental mechanical properties of various isotropic advanced composite materials that could be used in wind turbine blades. These materials exhibit homogeneity, meaning their density remains uniform throughout their volume. Additionally, they are assumed to be isotropic, possessing identical mechanical properties in all directions [15]. These isotropic properties were derived from homogenized composite behavior under multiaxial loading. This assumption is commonly used in preliminary finite element analysis (FEA) studies to reduce complexity and computational cost and is considered acceptable when directional dependencies are minor or when only global responses (e.g., deflection, stress distribution) are of interest [15]. For the basalt fiber composite, an equivalent plate theory was employed to represent the composite sections. This method homogenizes the laminate stack into an equivalent single-layer material with effective stiffness properties. This allows isotropic or quasi-isotropic modeling assumptions to be made [22,23].

However, in certain cases, anisotropic materials—specifically orthotropic materials—can also be considered. One such material of interest is bamboo, which has been formulated as a composite material (Table 2). Bamboo offers several key advantages, including low density, high sustainability, cost-effectiveness, rapid growth, and superior mechanical properties compared to many traditional woods. Its compatibility with composite manufacturing further enhances its potential as an alternative material for wind turbine blades [24,25]. A disadvantage is its tendency to absorb moisture, which impairs the fiber-matrix bonding and decreases the composite’s strength and stiffness. An alternative is bamboo composites supplemented with thermosetting polymers, which may resist moisture, though such technology is still under development [26]. Given the limited research on bamboo composites for large-scale blade skins, its application in this context warrants further investigation.

Table 2 presents the mechanical properties of a bamboo composite with a synthetic polymer matrix (epoxy)—an orthotropic material. The notations (x,y,z) in Table 2 correspond to the principal material axes of the Cartesian coordinate system of Figure 2. Unlike isotropic materials, orthotropic ones exhibit different mechanical behaviors depending on the direction of loading. For properties such as the Poisson’s ratios and density, experiments may be performed on real bamboo specimens by using strain gauges [24]. However, determining the rest of the mechanical properties of natural fiber composites is challenging. Bora et al. [25] applied a micromechanical approach to estimate the elastic properties of a bamboo-based composite, using a square representative volume element (RVE) with periodic boundary conditions. This method accounts for the composite’s repeating microstructure and enables a more accurate evaluation of its mechanical behavior compared to simpler analytical models.

## 3. IEA 15 MW Wind Turbine Blade Analysis

The IEA 15 MW wind turbine is designed for both fixed-bottom and floating offshore applications. In this study, the focus is on its floating base, as this configuration is particularly suitable for deployment in Greek waters. The key specifications of the wind turbine are presented in Table 3.

**Figure 5:**
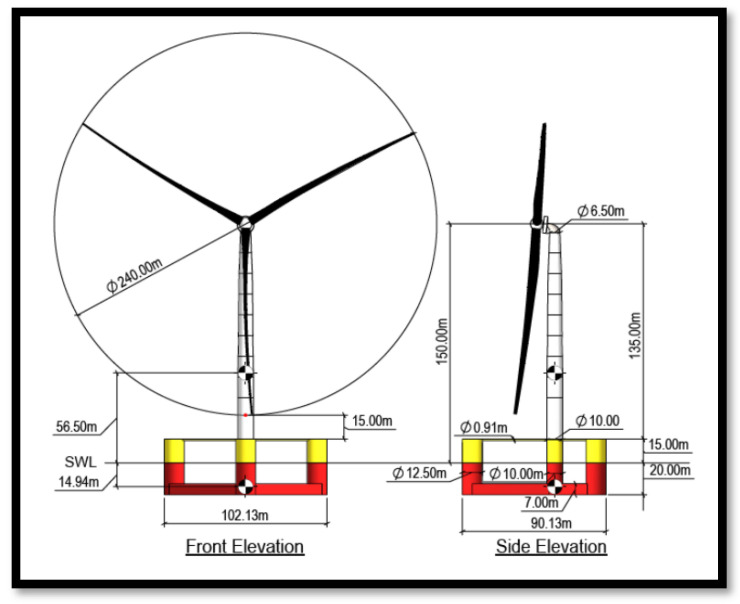
IEA 15 MW wind turbine dimensions [11].

Figure 5 provides an engineering drawing illustrating the wind turbine’s primary dimensions, along with its floating platform. As an IEC 61400 Class IB turbine, it is designed to withstand extreme wind speeds of up to 70 m/s [27].

The primary control mechanism of the wind turbine is pitch angle control, which regulates the blade pitch angle to optimize aerodynamic performance. Adjusting the pitch angle modifies the angle of attack, thereby controlling aerodynamic forces and influencing the power output. This regulation allows the torque to remain constant beyond the nominal operating point. Consequently, even as wind speeds increase, the electrical generator remains protected from overloading.

**Figure 6:**
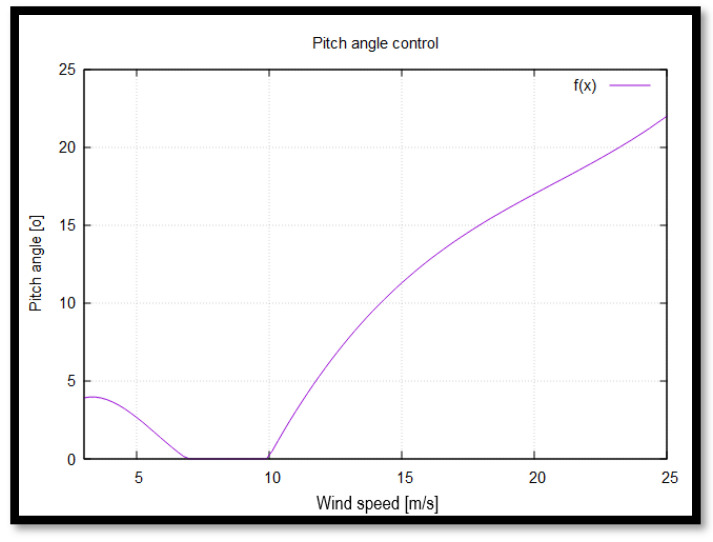
Wind speed $\left|\vec{V_{\infty }}\right|$ and pitch angle θ relationship.

Figure 6 illustrates the relationship between the wind speed $\left|\vec{V_{\infty }}\right|$ [m/s] and the pitch angle θ [°].

The operation of a wind turbine can be categorized into three distinct regions based on wind speed conditions (Figure 6). In Region 1, which corresponds to low wind speeds (3–7 m/s), the available wind intensity is insufficient to reach the turbine’s rated power. To maximize power capture, the pitch angle is adjusted to increase the generated torque.

Region 2 represents the rated power conditions, where the turbine operates close to its maximum designed power output. During this stage, the pitch angle remains at zero degrees to ensure optimal aerodynamic efficiency.

In Region 3, also known as the power limitation mode, the turbine’s control system actively adjusts the pitch angle to prevent overloading while maintaining maximum power output within safe operational limits. However, if wind speeds exceed an upper threshold, pitch angle adjustments alone are no longer sufficient to regulate power generation.

Beyond Region 3, when wind speeds exceed the cut-out limit, a dedicated braking system prevents the rotor from rotating. Simultaneously, the pitch angle is set to 90 degrees, fully engaging the feathered mode, ensuring minimal structural loading and protecting the turbine components from excessive stress.

**Figure 7:**
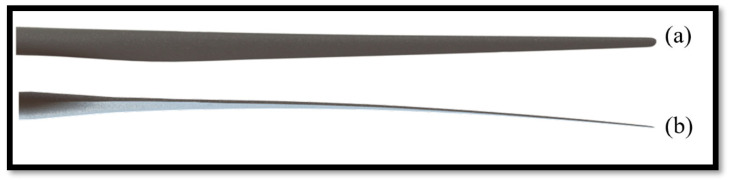
CAD representation of the (**a**) suction side and (**b**) trailing edge of the IEA 15 Wind Turbine blade [11].

Figure 7 illustrates the IEA 15 MW wind turbine blade in its CAD representation. The pronounced pre-bending curvature is clearly visible, a design feature that mitigates structural loads and reduces operational fatigue. Pre-bending is a critical consideration in offshore wind turbine blades, enhancing structural integrity and aerodynamic efficiency.

The primary geometric and physical properties of the IEA 15 MW wind turbine blade are summarized in Table 4. Other geometric characteristics, such as airfoil types and thickness, are presented in Table 5.

## 4. Weather Conditions of the Site

One of the first offshore wind farms in Greece is expected to be installed near eastern Crete, in Sitia. Given the location of the wind turbine, but with limited available wind resource data, estimations must be made based on meteorological studies and Weibull distribution models.

The Weibull distribution is widely employed in modeling failure probabilities of physical systems due to external factors. It is particularly relevant to wind energy applications, as its flexible nature allows it to model wind speed frequency distributions over the operational lifespan of a wind farm. Compared to direct experimental wind measurements, the Weibull distribution provides more reliable long-term predictions of wind potential. The probability density function for wind speed is given by [29]:

(1) $$f\left(u\right)=\frac{k}{c}\cdot {\left(\frac{\left|\vec{V_{\infty }}\right|}{c}\right)}^{k-1}\cdot e^{-{\left(\frac{\left|\vec{V_{\infty }}\right|}{c}\right)}^{k}}$$

where $\left|\vec{V_{\infty }}\right|$ is the wind speed, and *k* and *c* are parameters that form the final figure of the curves. Specifically, *k* is the shape parameter, which determines how variable the wind speed is. On the other hand, *c* is the scale parameter, which is related to the average wind speed. Determining these parameters requires statistical data, which is not fully available for the study site and thus must be estimated using empirical methods.

Wind speed measurements for meteorological or wind resource assessments are typically recorded over a year, at a height of 10 m, with the average wind speed calculated as:

(2) $$\bar{u}=\sum_{i=1}^{v}\frac{u_{i}}{v},i=1,2,3,\ldots ,v$$

For the Aegean Sea, the mean annual wind speed is reported to be 8.5–9.0 m/s, based on related studies conducted. Specifically, for the offshore region east of Sitia, the wind speed is estimated at 8.5 m/s. The site is also strategically advantageous, as it is free from major maritime traffic and has a relatively shallow seabed [30,31].

Since the IEA 15 MW wind turbine hub is located at 150 m (Figure 5), the wind speed at this height must be extrapolated using atmospheric boundary layer theory. The motion of air relative to the Earth’s rotation forms a boundary layer, influenced by surface roughness (e.g., sea or land). For practical wind speed calculations, the power law equation is applied:

(3) $$\frac{\left|\vec{V_{\infty }}\right|}{u_{ref}}={\left(\frac{z}{z_{ref}}\right)}^{a}$$

where *u_ref_* is the wind speed at reference height *z_ref_*, and *α* is an empirical exponent dependent on terrain characteristics. For this study, a value of *α* = 0.14, commonly used in wind energy literature, was selected [32].

The standard deviation of wind speed measurements is not available, but it is necessary for estimating the Weibull parameters *k* and *c*. Based on wind studies from the Aegean islands, the coefficient of variation (CV) for wind speed is estimated at 53.573%, derived from the average values of Mykonos and Lesvos. The CV, which indicates the variability of a dataset, is expressed as:

(4) $$CV=\frac{s}{\left|\bar{u}\right|}\cdot 100=\frac{s}{\bar{u}}\cdot 100\left[\%\right]$$

where *s* is the standard deviation of the wind speed measurements. Using *s* = 4.55 m/s for an average wind speed of 8.5 m/s, Bowden’s empirical relationships can be applied to determine the Weibull parameters [33].

(5) $$k≅{\left(\frac{s}{\bar{u}}\right)}^{-1.086}\overset{\left(4\right)}{\overbrace{=}}{\left(\frac{CV}{100}\right)}^{-1.086}$$

(6) $$c=\frac{\bar{u}}{\Gamma \left(1+\frac{1}{k}\right)}\left[m/s\right]$$

where *Γ* represents the Gamma function, which can be computed using numerical methods.

The calculated values are *k* = 1.97 and *c* = 9.59 m/s.

**Figure 8:**
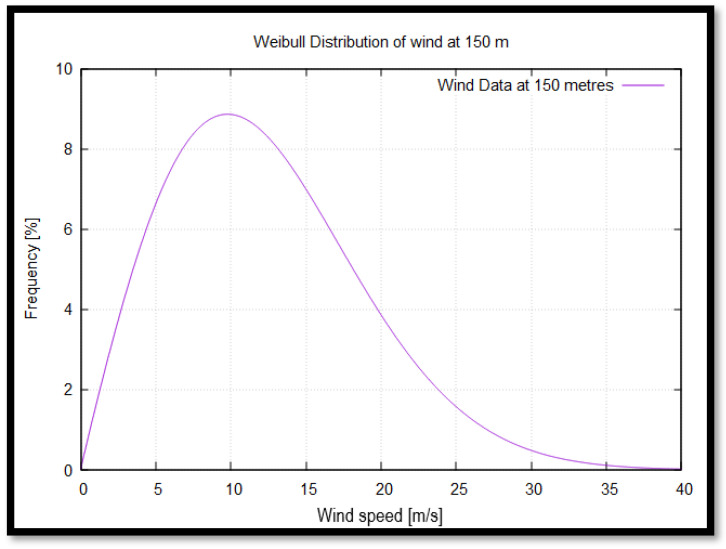
Weibull distribution of the wind velocity at 150 m above sea level.

From the Weibull distribution analysis and Figure 8, two key operational regions are identified:

- Rated operating conditions: The highest wind speed occurrence is around 10 m/s, with a probability of 8.5%, aligning closely with the rated wind speed of 10.59 m/s for the IEA 15 MW wind turbine (Table 3).
- Cut-out wind speed: Wind speeds exceeding 35 m/s have an almost negligible probability. At this threshold, the turbine enters feathering mode, where rotation ceases to ensure structural integrity. This speed remains well below the turbine’s design survival wind speed, confirming its operational safety (Table 3).

**Figure 9:**
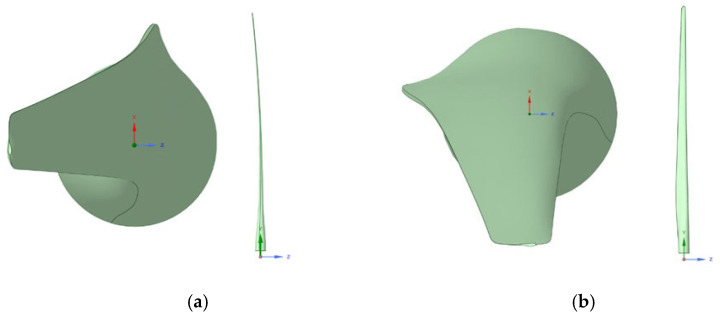
Configurations of the blade in the 3D environment. In configuration (**a**), the blade is in feathered mode. In configuration (**b**), the blade is operating at the rated conditions.

To simulate these two critical conditions, the wind velocity $\vec{V_{\infty }}$ is assumed to follow the +X-direction, and the turbine blade is positioned in a 3D computational environment in two configurations (Figure 9). In configuration (a), the blade is set in feathering mode at a 90-degree pitch angle relative to the wind flow, representing the extreme wind speed scenario (35 m/s wind speed). In configuration (b), the blade is positioned at a zero-degree pitch angle, corresponding to rated operating conditions (10.59 m/s wind speed).

## 5. Computational Fluid Dynamics Analysis

For the analysis of complex fluid–structure interactions and aerodynamic behavior, computational fluid dynamics (CFD) methods are employed. These methods utilize specialized software to discretize the computational domain using the finite volume method (FVM). The Navier–Stokes equations, which govern fluid motion, are then solved numerically at each discretized control volume. The objective is to achieve solution convergence, with the degree of accuracy defined by the user.

The fundamental governing equations in this study are [34]:

(7) $$\frac{\partial \rho }{\partial t}+div\left(\rho \overset{⃑}{u}\right)=0$$

(8) $$\rho \frac{D\overset{⃑}{u}}{Dt}=-grad\left(p\right)+div\left(\overset{⃡}{\tau }\right)$$

where *ρ* is the fluid density, *p* is the static pressure, $\overset{⃑}{u}$ is the velocity vector, and $\overset{⃡}{\tau }$ is the stress tensor. Equation (7) represents the continuity equation, ensuring mass conservation, while (8) corresponds to the momentum conservation equation, which describes the balance of forces in the fluid.

The simulations in this study were conducted using ANSYS Fluent© [35], a widely used CFD solver from the ANSYS© suite [35]. The analysis focuses on two steady-state flow conditions:rated operating conditions, for a wind speed of 10.59 m/s, and feathering mode, corresponding to extreme wind conditions at 35 m/s.

Given the relatively low wind velocity, the airflow is assumed to be incompressible. Table 6 summarizes the parameters selected for the flow modeling. Air is used as the working fluid, with standard ambient density and viscosity values applied. A constant temperature of 293.1 K (25 °C) is assumed, and thermal effects are neglected. As such, the influence of temperature on both the flow field and the composite material behavior is not considered in this study.

Although air properties vary slightly with altitude, at the wind turbine hub height of 150 m, the density and viscosity decrease by approximately 2% compared to sea level. This variation is considered negligible for the present analysis.

The *k-ω* SST (shear stress transport) turbulence model was selected for this study as it provides improved accuracy over previous models. While alternative turbulence models were tested, the *k-ω* SST formulation yielded results that closely aligned with expected physical behavior. The *k* term represents the turbulent kinetic energy, while *ω* denotes the specific dissipation rate. Prior research indicates that the *k-ω* SST model effectively captures transitional flow phenomena at the fluid–wall interface, making it well-suited for wind turbine blade simulations [15,34,36].

**Figure 10:**
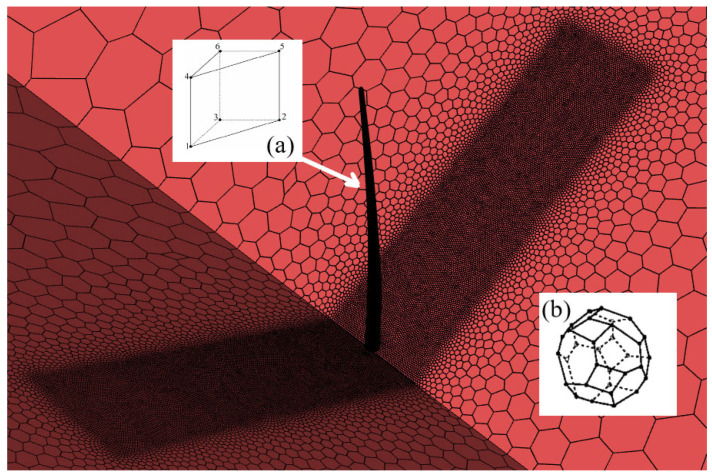
Fluid domain mesh. Prismatic elements (**a**) are used for the boundary layer along the blade. Polyhedral elements (**b**) are used for the rest of the flow domain.

The computational mesh of the fluid region consists of polyhedral elements, providing an efficient and precise representation of the flow field (Figure 10). To model transient phenomena, such as boundary layer formation on the blade, pentahedral elements are employed. Monitoring the boundary layer is crucial, as it influences flow transition from laminar to turbulent and can lead to flow separation, increasing drag and reducing aerodynamic efficiency. Pentahedral elements, as linear prismatic finite elements, form thin layers within the mesh, allowing for an accurate and efficient representation of boundary layer dynamics (Figure 10).

**Figure 11:**
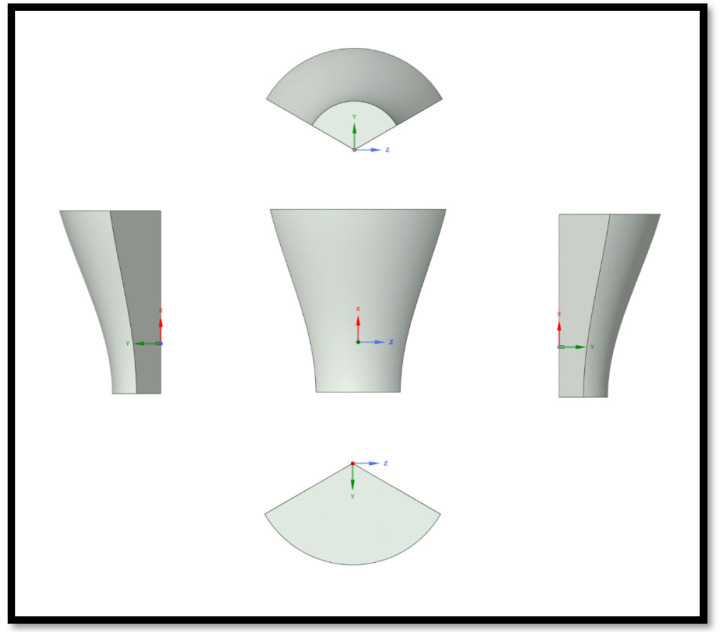
Views of the flow domain geometry.

**Figure 12:**
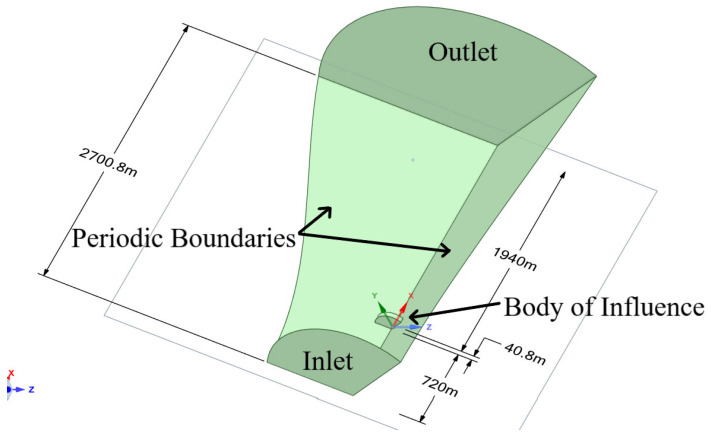
Size of the flow domain and its boundaries. The geometric model of the blade is located within the body of influence.

The flow domain is designed as an elongated cylindrical region, allowing for the full development of the airflow [14,15] and the prevention of reverse flow (Figure 11). The total length of the domain is approximately 2.7 km, similar to the strategy of Zhang et al. [14]. The inlet radius is 720 m, and the outlet radius is 1.5 km. At a distance of six lengths of the blade (720 m), the blade is positioned in relation to the entrance (inlet). The outlet is approximately over a length of sixteen blades (1940 m) from the blade model (Figure 12).

To optimize computational efficiency, the simulation domain represents a 120-degree sector of the rotor, corresponding to one-third of a full circle. These set of boundaries are called periodic, and they enforce the condition that the flow variables (e.g., velocity, pressure) on one periodic face are mapped to the opposite face with a rotational transformation. In cylindrical coordinates, this can be expressed as:

(9) $$\vec{u}\left(r,\theta ,z\right)=\vec{u}\left(r,\theta +\Delta \theta ,z\right)$$

where $\vec{u}$ is the velocity vector, and Δθ=120° and $\left(r,\theta ,z\right)$ are the cylindrical coordinates. By employing periodic boundary conditions, the entire rotor can be modeled using a single blade, significantly reducing computational cost. However, this simplification may overlook the potential effects of the interactions between the three blades and their influence on the wind flow dynamics.

The boundary conditions applied in the simulation are as follows:

- Inlet: Uniform velocity of 10.59 m/s or 35 m/s in the X+-direction, with a turbulence intensity of 5% and a turbulent viscosity ratio of 10.
- Outlet: Static pressure set to 1 atm.
- Wind turbine surface: No-slip condition applied to capture near-wall effects.
- Fluid region walls: Periodic boundary conditions to replicate full rotor operation. The axis of rotation is the global X-axis.
- General conditions:
  ○ For rated operating conditions: rotational velocity of ω = 0.79 rad/s in the X+-direction.
  ○ For feathering mode: rotational velocity of ω = 0, corresponding to a stationary blade configuration.

Based on the data in Table 7 and Table 8, the quality of the computational mesh is adequate for both cases.

For the case of the stationary blade modeled as a rigid wall ($\vec{V_{\infty }}$ = 35 m/s), a full mesh study was not conducted. However, incremental mesh refinement showed negligible changes in results, indicating mesh independence. This setup lacks the unsteady flow effects present in rotating simulations, reducing modeling complexity and sensitivity to mesh resolution, which justifies the lower element count.

The quality of the simulation results is primarily validated against reference data provided by the developers of the wind turbine model, as well as findings from other researchers [11,12,13,14]. For both steady-state cases, convergence was achieved after approximately 2000 iterations on average. Specifically, the simulations were considered converged when the residuals for continuity, velocity (in all three directions), and turbulence (k, ω) dropped below 10^−6^. Due to differences in mesh density (as shown in Table 7 and Table 8), computational time varied significantly between the two cases, with the finer mesh requiring substantially more time to converge.

A mesh sensitivity analysis was conducted for the rotating blade case under rated conditions (V_∞_ = 10.59 m/s), comparing coarse (307,741 elements) and fine (3.1 million elements) meshes (Table 8). Key mesh metrics improved with refinement (orthogonal quality and skewness), and aerodynamic outputs, such as thrust and flapwise bending moment, differed by far less than 10% with available data. Beyond three million elements, the computational cost increased significantly, with minimal gains in accuracy.

A key measure of accuracy is the axial thrust generated by the wind turbine rotor during operation. In the fluent computing environment, this thrust can be approximated as the net force exerted on the blade surfaces in the direction of the wind (axial direction, X-direction, Figure 11). Exact data for the rotor thrust ${\vec{T}}_{rotor}$ at rated speed is unavailable. However, linear interpolation can be applied using data from other wind speeds, as shown in Table 9.

For a wind speed of 10.59 m/s, linear interpolation can be performed as follows:

(10) $$\frac{10.6584-10.59}{10.6584-10.2096}=\frac{2.4473-{\vec{T}}_{rotor}}{2.4473-2.2644}\Leftrightarrow \;\Leftrightarrow {\vec{T}}_{rotor}=2.4473-0.0279=2.4194\;MN$$

Therefore, assuming that the thrust is evenly distributed over the three blades:

(11) $$\vec{T}=\frac{{\vec{T}}_{rotor}}{3}=0.806\;MN$$

Another measure of evaluation of results is the flapwise bending moment exerted by the wind (Figure 4). Similar to before, for a wind speed of 10.59 m/s and the data from Table 10:

(12) $${\vec{M}}_{flap}=64.4131\;MNm$$

By comparing the values obtained from Equations (11) and (12) with the simulation results presented in Table 8 and the findings of previous studies [13,14,37], it is determined that the calculated thrust and bending moment exhibit good agreement with established data. Specifically, the bending moment shows a maximum deviation of approximately 9.60%, while the thrust deviation reaches 8.75%. These discrepancies are considered acceptable given the limitations imposed by mesh resolution [14] and variations in computational solvers, which can introduce differences in numerical results [38]. In particular, the current study performed a mesh sensitivity analysis, demonstrating that no significant changes in the results occurred with an increase in mesh resolution. This suggests that mesh resolution was not a significant source of error. Differences in solver settings, including turbulence models, boundary conditions, and time-stepping schemes, can introduce variability in results. Additionally, variations in computational methods, such as turbulence model selection and numerical discretization, can further contribute to discrepancies in the results.

Since experimental data for stress distributions (e.g., strain gauge measurements from similar blades) were unavailable for direct comparison, the numerical results presented here are considered reasonable. However, validation of stress distributions with experimental data remains a valuable direction for future work, as it would help refine the accuracy of the simulations, especially for cases involving complex loading conditions.

**Figure 13:**
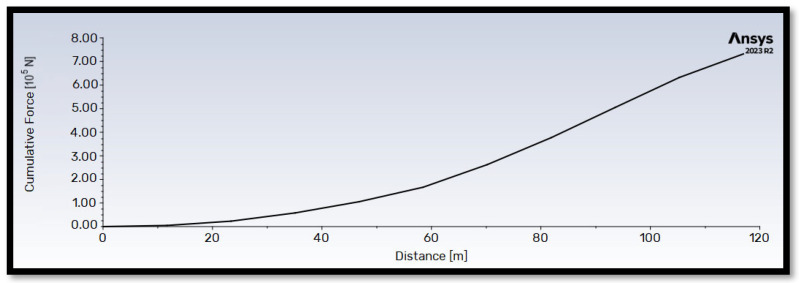
Cumulative thrust $\vec{T}$ along the length (distance from the center of rotation) of the blade. The wind velocity has a value of $\vec{V_{\infty }}$ = 10.59 m/s.

**Figure 14:**
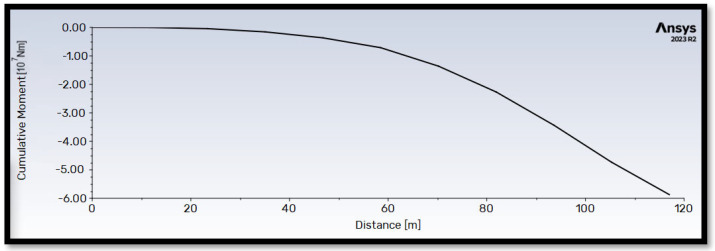
Cumulative flapwise bending moment ${\vec{M}}_{flap}$ along the length (distance from the center of rotation) of the blade. The wind velocity has a value of $\vec{V_{\infty }}$ = 10.59 m/s.

Figure 13 and Figure 14 illustrate the cumulative thrust and flapwise bending moment along the blade length for operation at the rated wind velocity ($\vec{V_{\infty }}$ = 10.59 m/s). Both quantities exhibit a generally proportional relationship with blade length. Notably, the flapwise bending moment reaches a maximum value approximately 100 times higher than the thrust force.

**Figure 15:**
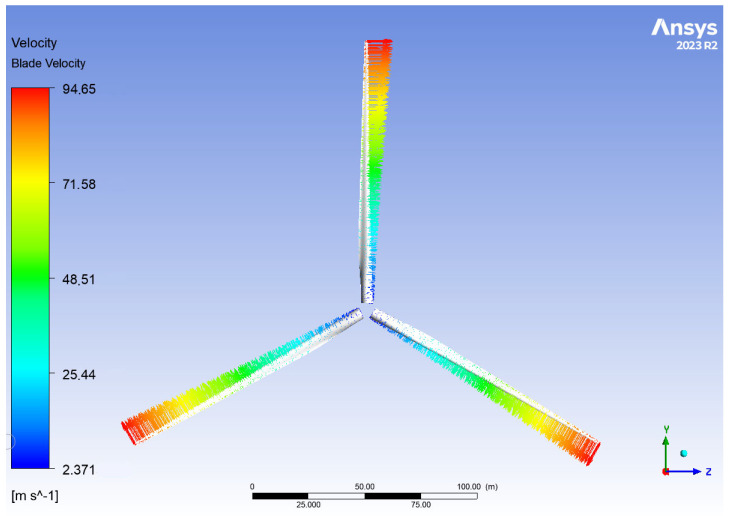
Rotational velocity of the rotor for operation at the rated wind speed $\left|\vec{V_{\infty }}\right|$ = 10.59 m/s.

Figure 15 depicts the rotational velocity of the rotor. According to the no-slip condition, the fluid velocity matches the blade velocity at the point of contact. The observed range of rotational velocity aligns with expected values [11].

**Figure 16:**
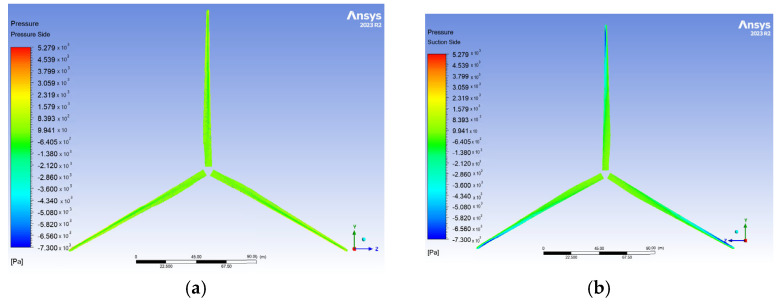
Pressure contours on the blade surface for $\vec{V_{\infty }}$ = 10.59 m/s. The pressure side is depicted in (**a**), while the suction side is depicted in (**b**).

The pressure distribution on the blades in Zhang et al. [14] ranges from −10 kPa to 4.82 kPa, while the results of this study (Figure 16) exhibit a comparable range, specifically −7.3 kPa to 5.28 kPa for the dense mesh, corresponding to a 9.9% deviation. As expected, the leading edge and pressure side experience higher pressures compared to the trailing edge and suction side.

**Figure 17:**
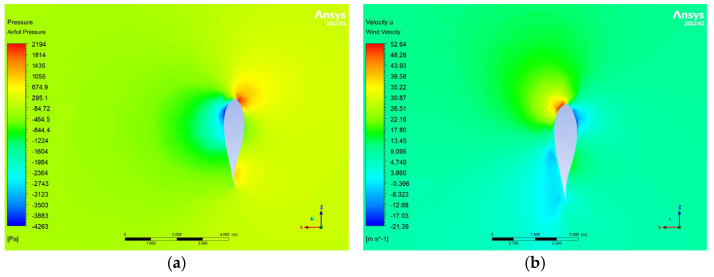
(**a**) Pressure contour of the airflow at *r/R* = 0.67 for $\vec{V_{\infty }}$ = 10.59 m/s. (**b**) Flow velocity contour at *r/R* = 0.67 for $\vec{V_{\infty }}$ = 10.59 m/s.

The wind velocity results under rated conditions align with those reported by Zhang, Y. et al. [14], with a maximum deviation of 5% at peak wind speed (Figure 17).

**Figure 18:**
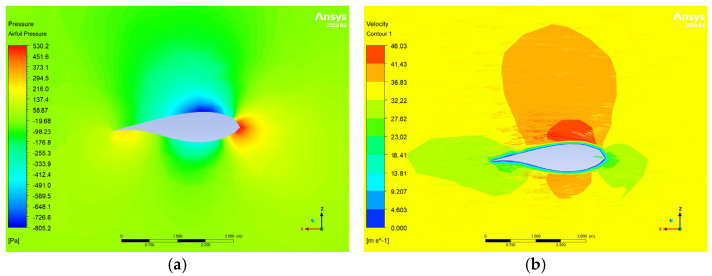
(**a**) Pressure contour of the airflow for $\vec{V_{\infty }}$ = 35 m/s. (**b**) Flow velocity contour for $\vec{V_{\infty }}$ = 35 m/s.

Figure 18 illustrates the effect of placing the blade in feathered mode. Despite an increase in wind speed, the pressure decreases by a factor of 10, demonstrating the effectiveness of this configuration in reducing aerodynamic loads. Given the adequacy of the CFD results, FSI analysis was subsequently employed for computational structural evaluation.

## 6. Computational Structural Analysis

For the analysis of complex static problems, the finite element analysis (FEA) method is employed. Similar to CFD methods, FEA utilizes computational software to discretize the examined structure into a mesh of finite elements. The governing equations are then numerically approximated and solved for each element using appropriate numerical analysis techniques.

In ANSYS© Mechanical [39], the displacement-based finite element formulation is applied, meaning that the equations are solved with respect to nodal displacements. For static analysis, the fundamental equations to be solved are as follows [40]:

(13a) $$\nabla \cdot \vec{\sigma }+\vec{F}=0\;or$$

(13b) $${\tilde{F}}^{e}={\tilde{k}}^{e}\cdot {\tilde{q}}^{e}$$

(14) $$\tilde{q}=\tilde{N}\cdot {\tilde{q}}^{e}$$

(15) $$\tilde{\varepsilon }=\tilde{B}\cdot {\tilde{q}}^{e}$$

(16) $$\tilde{\sigma }=\tilde{D}\cdot \tilde{\varepsilon }$$

Equation (13a) represents the equilibrium condition of the system, while Equation (13b) is obtained after discretization into finite elements, where ${\tilde{q}}^{e}$ is the element displacement matrix and ${\tilde{k}}^{e}$ is the element stiffness matrix. Equation (14) defines the relationship between the overall body displacement $\tilde{q}$ and the element displacements using the shape functions $\tilde{N}$, which depend on the selected finite element type. Equation (15) links strain $\tilde{\varepsilon }$ to nodal displacements through the strain–displacement matrix $\tilde{B}$. Finally, Equation (16) represents Hooke’s law, where stress $\tilde{\sigma }$ is related to strain $\tilde{\varepsilon }$ via the material stiffness matrix $\tilde{D}$.

For an isotropic material, the stiffness matrix $\tilde{D}$ takes the form:

(17) $$\tilde{D}=\frac{E}{\left(1+v\right)\left(1-2v\right)}\left[\begin{array}{cccccc} 1-v & v & v & 0 & 0 & 0\\ v & 1-v & v & 0 & 0 & 0\\ v & v & 1-v & 0 & 0 & 0\\ 0 & 0 & 0 & \frac{1-2v}{2} & 0 & 0\\ 0 & 0 & 0 & 0 & \frac{1-2v}{2} & 0\\ 0 & 0 & 0 & 0 & 0 & \frac{1-2v}{2} \end{array}\right]$$

where E is the modulus of elasticity, and v is Poisson’s ratio. For orthotropic materials, the stiffness matrix $\tilde{D}$ differs due to direction-dependent material properties and is no longer symmetric.

In this study, small displacements are assumed. The materials used are considered homogeneous and to remain within the linear elastic region. The solver utilized is APDL© (ANSYS Parametric Design Language) [39].

At the time of this study, an original CAD model suitable for structural analysis of the IEA 15 MW blade was not available [11]. Instead, the analysis utilized the parametric CAD model developed by Mendoza, ASE et al. [41], which is publicly accessible. This model incorporates surface elements as internal shear webs. It includes only the blade skin and shear webs, omitting other important structural components typically present in wind turbine blades, such as the spar caps. Most notably, spar caps are responsible for carrying a significant portion of the bending loads in large blades, and their absence likely leads to an underestimation of blade stiffness and an overestimation of stress in the remaining components. Additionally, the simplified modeling of shear webs as surface elements may alter the load transfer mechanisms within the blade. As a result, the stress distribution and deformation patterns observed in the simulations may not fully represent the actual behavior of the complete blade structure, limiting the accuracy of the structural performance predictions under operational loads.

The procedure for properly orienting the blade, considering both operational cases, follows the approach illustrated in Figure 9. This step is crucial for the accurate transfer of CFD data to the FEA model using the fluid–structure interaction (FSI) method. For each surface type, including the blade skin and shear webs, the appropriate thickness is assigned based on the values provided in Table 5.

**Figure 19:**
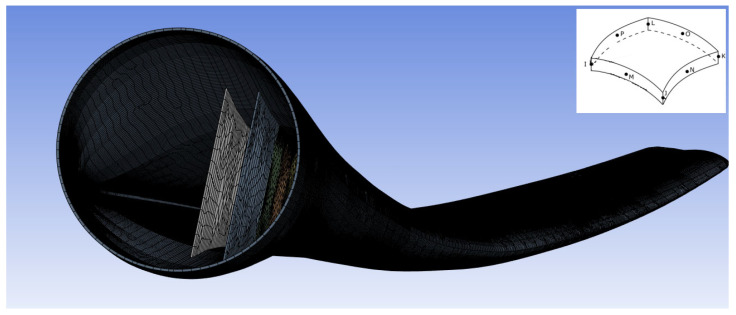
Mesh of the geometric blade model for static structural analysis. In the upper right corner, the SHELL281 element is depicted. It is a second-order element with 8 nodes.

Second-order shell elements (ANSYS© SHELL281 [39]) are chosen for the static structural analysis (Figure 19). These elements are well-suited for modeling thin-walled structures, making them appropriate for the blade’s thickness, as specified in Table 5. Additionally, they ensure high mesh quality while maintaining computational efficiency, as indicated by the element count in Table 11.

As with the CFD analysis, only one blade is examined, thus excluding the hub and tower. This approach disregards dynamic effects such as oscillations from the floating base. Their inclusion was deemed unnecessary given the already simplified nature of the model. The CAD model used for the structural analysis differs from the model used in the CFD analysis, and this discrepancy could introduce minor deviations in the results due to the FSI method.

Environmental effects such as solar radiation and marine conditions were also not considered. While these factors significantly impact material degradation and blade longevity, this study focuses solely on two steady-state flow conditions, which do not capture the full complexity of real-world environmental influences.

In this one-way FSI analysis, the steady-state aerodynamic loads obtained from the CFD simulations were applied as input forces for the static structural analysis, thus serving as boundary conditions. For higher accuracy, a two-way FSI analysis—where structural deformation affects the aerodynamic loading—would be required. However, this approach is often avoided due to the significant computational resources it demands [15,16,17].

The blade is fixed at the root, with reaction forces and moments applied at a reference point 3 m from the root cross-section. This setup simulates the presence of the hub, which is not explicitly included in the CAD model. The root boundary condition prevents unintended displacements, ensuring accurate solver calculations. The hub is modeled as a rigid body, serving as the reference point for displacement constraints.

For rated operating conditions, the rotation of the blade is simulated by applying an angular velocity of +0.79 rad/s in the direction of the wind.

To assess the structural integrity of the blade under aerodynamic loading, the equivalent von Mises stresses were calculated and compared to the ultimate tensile strength of the materials. The von Mises stress is determined using the following equation:

(18) $$\sigma_{eq}=\sqrt{\sigma_{x}^{2}+\sigma_{y}^{2}+\sigma_{z}^{2}-\sigma_{x}\sigma_{y}-\sigma_{y}\sigma_{z}-\sigma_{z}\sigma_{x}+3\left(\tau_{xy}^{2}+\tau_{yz}^{2}+\tau_{zx}^{2}\right)}\;[Pa]$$

where *σ* represents the normal stresses and *τ* represents the shear stresses. The maximum tensile strength values for the materials are provided in Table 12.

The ultimate tensile strength values were used in the absence of data on yield strength, as the materials were considered isotropic. Thus, the von Mises criterion was applied to assess the risk of material failure. For bamboo, an orthotropic material, shear strength values ranging from 40 MPa to 80 MPa were considered. For other conventional materials, such as glass fiber reinforced polymer (GFRP), shear strength values typically exceed 95 MPa [44,45].

Five material configurations are analyzed, as detailed in Table 1 and Table 2. In all cases, the shear webs are composed of carbon fiber composites, while five different materials are evaluated for the blade skin.

**Figure 20:**
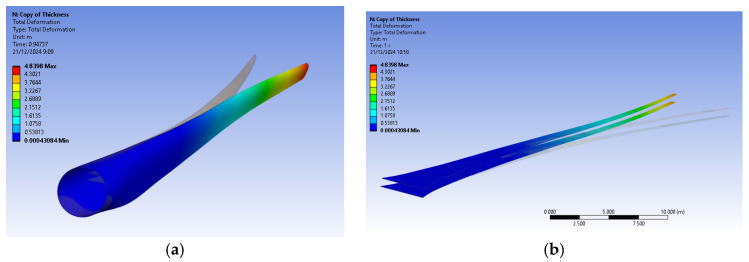
Displacement of (**a**) the blade skin and (**b**) the shear webs. $\vec{V_{\infty }}$ = 10.59 m/s, for bamboo composite blade skin.

**Figure 21:**
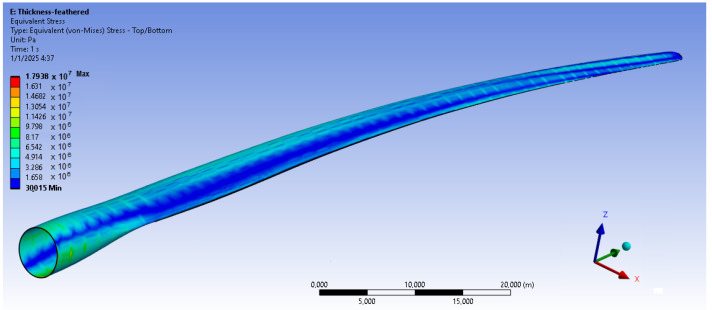
Equivalent von Mises stresses in the case of the bamboo-based blade skin. Higher von Mises stresses are present near root of the blade. $\vec{V_{\infty }}$ = 35 m/s.

**Figure 22:**
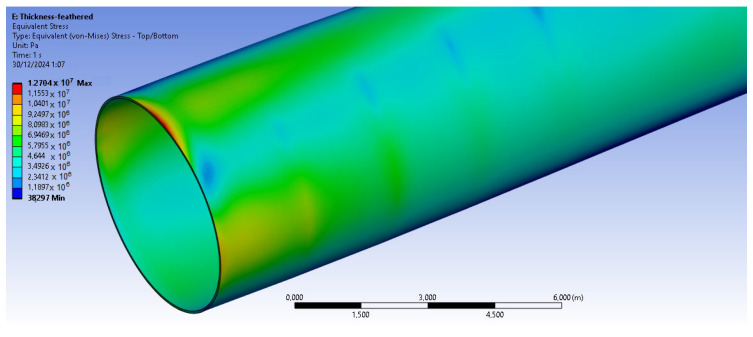
Close up of the blade, for the equivalent von Mises stresses in the case of the basalt fiber-based blade skin. $\vec{V_{\infty }}$ = 35 m/s.

**Figure 23:**
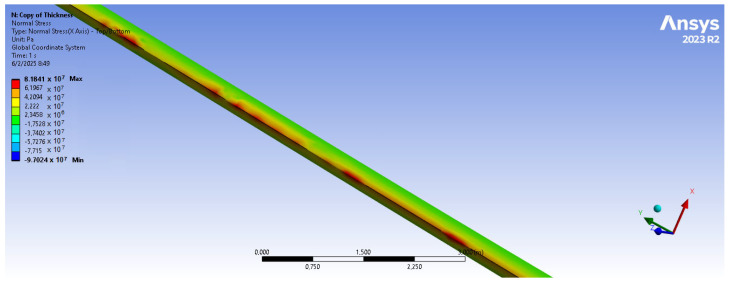
Normal stresses (X-axis) for bamboo-based blade skin. Higher normal stresses are present near the leading edge of the blade. $\vec{V_{\infty }}$ = 35 m/s.

**Figure 24:**
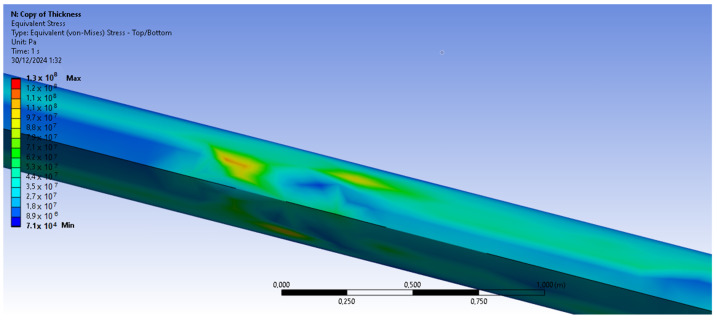
Equivalent von Mises stresses in the case of the bamboo-based blade skin. Higher von Mises stresses are present near the leading edge of the blade. $\vec{V_{\infty }}$ = 10.59 m/s.

**Figure 25:**
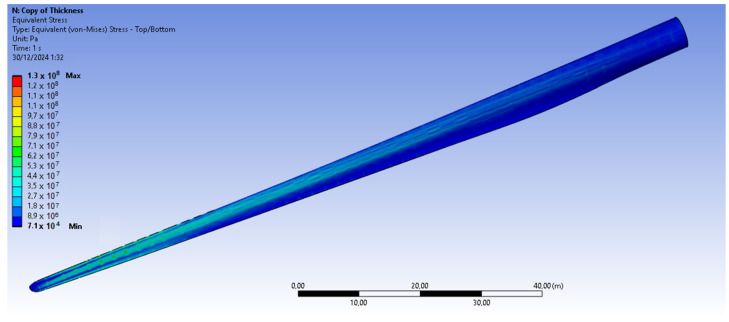
Equivalent von Mises stresses in the case of the bamboo-based blade skin. Pressure side of the blade. Higher von Mises stresses are present close to the blade tip. $\vec{V_{\infty }}$ = 10.59 m/s.

**Figure 26:**
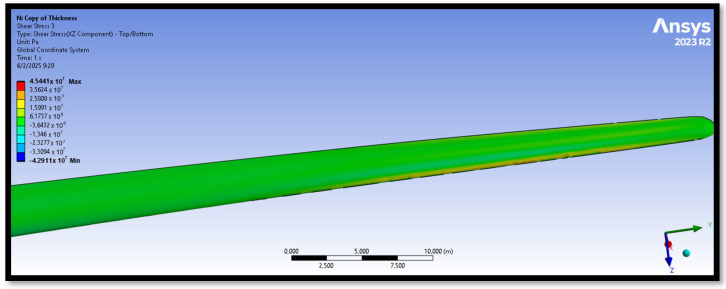
Shear stresses (XZ plane) in the case of the bamboo-based blade skin. The XZ plane corresponds to the leading edge, as the blade is spinning ($\vec{V_{\infty }}$ = 10.59 m/s).

The bamboo-based composite exhibits the highest displacement and stress values (Figure 20). When the blade is feathered, the root section experiences the greatest loading (Figure 21 and Figure 22). But under rated operating conditions, the highest stress concentrations occur at the blade tip and leading edge (Figure 23, Figure 24, Figure 25 and Figure 26). The normal stresses are lower in magnitude compared to the equivalent von Mises stresses, making the latter more suitable for assessing material performance and structural integrity (Figure 23). Shear stresses reach their peak on the XZ plane, likely due to the rotational motion of the blade, with maximum values concentrated near the leading edge (Figure 26).

## 7. Results and Discussion

We have already mentioned, that bamboo-based composite exhibits the highest displacement and stress values (Figure 20). However, all blade skin materials display similar overall stress and displacement distributions, meaning that Figure 20, Figure 21, Figure 22, Figure 23, Figure 24, Figure 25 and Figure 26 are representative of all configurations. Maximum equivalent von Mises stresses, displacements, and shear stresses for each scenario are summarized in Table 13.

To assess the validity of the results, a comparison is made with existing literature. A study by Theotokoglou and Xenakis [15] reports equivalent von Mises stress distributions of a similar order of magnitude, as well as comparable locations for stress maxima. Given that their analysis was conducted for smaller blades under similar environmental conditions, it is reasonable that the present study yields higher stress and deformation values. Notably, under rated conditions, the maximum equivalent von Mises stresses in their study are approximately 30% lower than those obtained in this work.

The magnitude of the computed stress values is consistent with those presented by Mendoza N et al. [13] and Zhang et al. [14]. In their investigations, the maximum von Mises stresses reached values in the range of hundreds of MPa, aligning with the findings of this study. Furthermore, the total blade displacement was found to be significant, approaching 5 m. According to Mendoza N. et al. [13], such displacements do not pose a risk of tower strikes. However, their studies focus on transient phenomena, which can lead to variations in the magnitude and location of maximum stress and displacement. Additionally, the materials analyzed in their work were predominantly orthotropic, whereas the present study assumes isotropic behavior for most materials. Another distinction is that the shear webs in this work are composed of carbon fiber, whereas previous studies [11] utilized glass fiber-reinforced composites. The selection of carbon fiber enhances structural rigidity, reducing total displacement but increasing mass.

The equivalent von Mises stresses exhibit a direct correlation with the modulus of elasticity *E* of most materials, with the exception of the bamboo composite, which is orthotropic. While the maximum equivalent stresses generally follow expected trends, total displacement is not strictly proportional to the elastic modulus or stress levels. Additionally, all materials demonstrate similar maximum shear stress values.

Based on the ultimate tensile strength data (Table 12), most materials can be deemed structurally viable for offshore wind turbine blades. However, bamboo-based composites present a higher risk of fracture and permanent deformation, as the maximum stresses approach their tensile strength limits (Table 13). Furthermore, bamboo exhibits relatively low shear strength compared to alternative materials, which may compromise long-term structural integrity.

**Figure 27:**
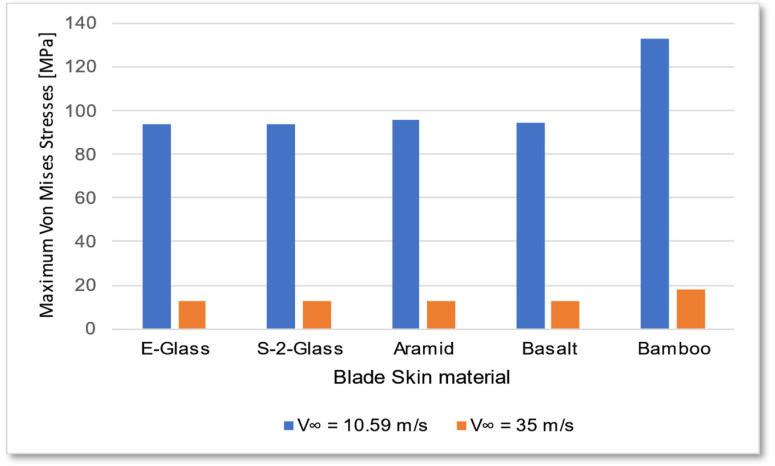
Maximum equivalent von Mises stresses for each blade skin material scenario.

**Figure 28:**
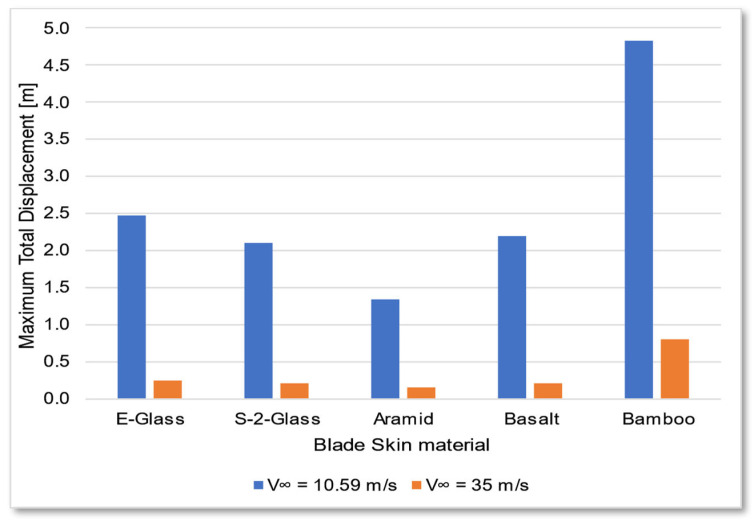
Maximum total displacement for each blade skin material scenario.

The data in Table 13 are visually represented in Figure 27 and Figure 28. Notably, transitioning the blade into feathered mode significantly reduces the load, even as the intensity of the wind increases.

Carbon fiber was included in the study, specifically as the material used for the shear webs across all blade skin configurations, consistent with the IEA 15 MW reference design. For this reason, carbon fiber was not separately evaluated as a skin material, as doing so would have introduced redundancy without significant added insight. The von Mises stress results for the shear webs made of carbon fiber are implicitly reflected in the overall structural analysis but were not individually tabulated, as the focus was on the comparative performance of the blade skin materials.

**Figure 29:**
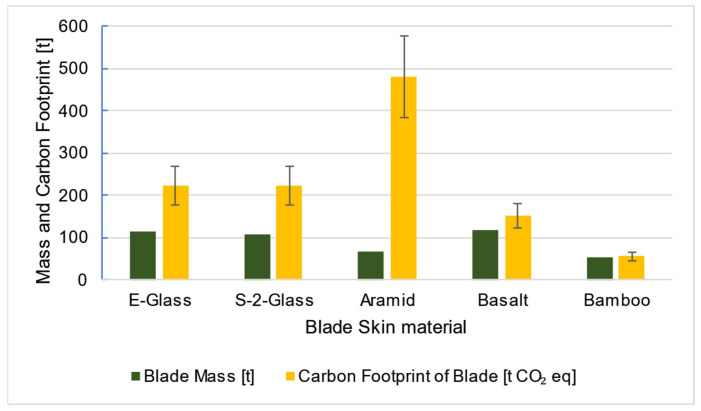
Comparison of all blade skin material scenarios in regard to the mass and carbon footprint of each wind turbine blade. The error bars correspond to an uncertainty of 20%. CO_2_ refers to carbon dioxide emissions.

**Figure 30:**
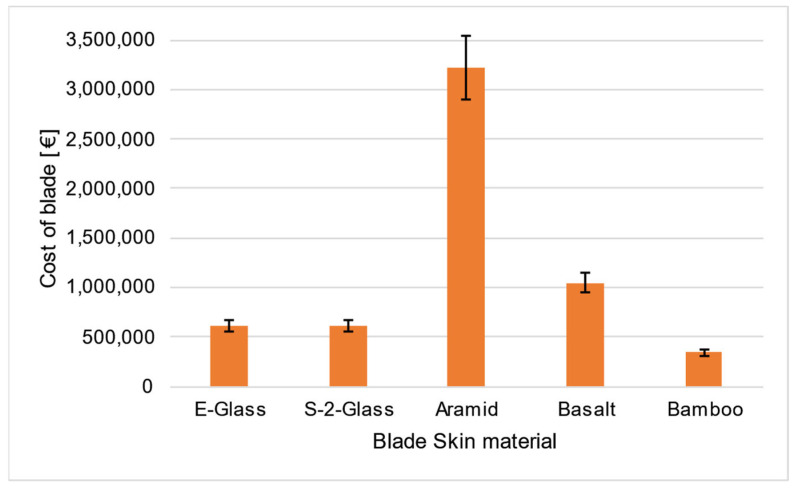
Comparison of all blade skin material scenarios in regard to the material cost of each wind turbine blade. The error bars correspond to an uncertainty of 10%.

Based on the simulation results, the blade mass was estimated for each material scenario. By integrating approximate emission factors [45,46,47] and cost per unit mass data [46,48,49] for each material, a comparative assessment was conducted in terms of both carbon footprint and economic feasibility. To account for variability in the data, an uncertainty margin of 20% was applied to the carbon footprint estimates (Figure 29) and 10% to the cost estimates (Figure 30), based on the range of values reported in the literature.

As shown in Figure 29, basalt fiber-based composites emerge as a promising ecological alternative, though density reduction or material redistribution may be necessary to optimize performance. Aramid fiber composites offer excellent mechanical properties but are costly, as shown in Figure 30. However, a hybrid composite combining aramid and basalt fibers could mitigate both financial and environmental impact. Glass fiber composites remain a widely used and reliable choice, with various formulations available to meet specific design requirements—such as low-density orthotropic materials used in the IEA 15 MW blade.

Natural fiber composites, however, are unsuitable for offshore wind turbines due to excessive deformation and structural failure risks. Specifically, the von Mises stress (133 MPa) reaches the maximum tensile strength, while the presented shear stress surpasses the reported shear strength limit (40 MPa), indicating a high probability of failure. Additionally, marine durability remains uncertain, making them impractical for large-scale offshore applications. Nevertheless, natural fiber composites may still be viable for smaller, domestic wind turbines, where their recyclability, low cost, and reduced environmental impact provide advantages.

## 8. Conclusions

This study focuses on the computational analysis of the IEA 15 MW wind turbine blade, with an emphasis on evaluating various composite materials. The research aligns with national plans for the development of floating offshore wind turbines, which feature large-diameter rotors requiring meticulous material selection to optimize performance, durability, and cost-effectiveness.

The material selection was categorized into two distinct groups. For the shear webs, carbon fiber composite was utilized, while the blade skin was examined using multiple options, including E-glass, S-2 glass, Aramid fiber-based composite, basalt fiber-based composite, and natural fiber composite (bamboo). A computational study was conducted using ANSYS© [35,39] to assess the viability of these materials, particularly focusing on the application of emerging and environmentally sustainable options. The aerodynamic geometry of the blade was derived from original sources, while additional publications provided data for static structural modeling.

Following aerodynamic analysis, a static structural evaluation was conducted, incorporating the derived aerodynamic loads into the stress analysis model. Equivalent von Mises stresses, shear stresses, and displacements were determined. The material combinations were assessed based on production cost, environmental impact, and mechanical performance. The findings revealed a trade-off between economic and environmental benefits and structural integrity. Natural fiber composites, while cost-effective and environmentally sustainable, exhibited performance limitations, reaching the threshold of permanent deformation and failure. Other materials increased the overall blade mass, negatively affecting aerodynamic efficiency. Basalt fiber composites emerged as a promising alternative due to their high resistance to corrosion from seawater and ultraviolet exposure, as well as their strong mechanical properties.

The findings support the integration of alternative composite materials, such as basalt and bamboo fibers, in wind turbine manufacturing to address cost and sustainability concerns.

The implementation of a two-way FSI model would enable a more dynamic assessment of blade deformation under aerodynamic loads. Additionally, modeling the tower, hub, and floating base would provide a more comprehensive understanding of wave-induced effects on structural behavior. The study of transient phenomena, such as fluctuating wind speeds and wave interactions, could further enhance simulation accuracy. Expanding material comparisons and testing various material combinations would offer deeper insights into optimal design strategies. Future work could also explore multi-material configurations or optimization studies to evaluate the strategic placement of high-performance fibers while maintaining cost-effectiveness. Finally, a fatigue analysis based on Weibull probability distributions could provide critical data on blade lifespan under specific environmental conditions, such as those prevalent in the Aegean Sea.

## Figures and Tables

**Table 1:** Mechanical properties of isotropic composites [15,22,23].

Material	E-Glass Fiber Composite	Aramid Fiber Composite	Carbon Fiber Composite	S-2 Glass Fiber Composite	Basalt Fiber Composite
Fiber type	Glass fiber	Aramid fiber	Carbon fiber	Glass fiber	Basalt fiber
Application	Blade skin	Blade skin	Shear webs	Blade skin	Blade skin
Density *ρ* [kg/m^3^]	2568.70	1439.30	1320.37	2460.74	2650
Modulus of elasticity *E* [GPa]	72.4	112	138	86.9	86
Poisson ratio *v*	0.20	0.36	0.3	0.23	0.28
Shear Modulus *G* [GPa]	30	7	5.7	35	33.59

**Table 2:** Mechanical properties of anisotropic (orthotropic) material—bamboo composite [24,25].

Material	Bamboo
Fiber type	Natural fiber
Application	Blade skin
Density [kg/m^3^]	1120
E_x_ [GPa]	22.37
E_y_ [GPa]	10
E_z_ [GPa]	10
G_xy_ [GPa]	4.30
G_xz_ [GPa]	4.30
G_yz_ [GPa]	3.30
v_xy_	0.35
v_xz_	0.35
v_yz_	0.25

**Table 3:** IEA 15 MW wind turbine basic specifications [11,27].

Characteristic	Value	Units
Rated power	15	MW
Specific power	332	W/m^2^
Number of blades	3	-
Cut-in wind speed	4	m/s
Cut-out wind speed	25	m/s
Rated wind speed	10.59	m/s
Rated rotational speed	0.79	rad/s
IEC Class 61400	IB	-

**Table 4:** Primary geometric characteristics of the IEA 15 MW wind turbine blade [11].

Characteristic	Value	Units
Blade length	117	m
Root diameter	5.20	m
Length of root cylinder	3.54	m
Max chord	5.77	m
Blade mass	65,250	kg
Blade center of mass	26.80	m

**Table 5:** Geometry and blade thickness along length [28].

Airfoil	Radius Ratio (r/R) [%]	CAD Geometry Length [m]	Blade Skin Thickness [m]	Shear Web Thickness [m]
SNL-FFA-W3-500	15	20.55	0.056	0.040
FFA-W3-360	24.51	31.67	0.0485	0.036
FFA-W3-330blend	32.88	41.47	0.042	0.032
FFA-W3-301	43.91	54.37	0.033	0.027
FFA-W3-270blend	53.76	65.90	0.0256	0.023
FFA-W3-241	63.82	77.67	0.017	0.018
FFA-W3-211	80.11	96.73	0.007	0.012

**Table 6:** Fluid mechanical analysis options.

Flow–viscosity model	K-omega (k-ω) (2 equations)
K-Omega model	Shear stress transport (SST)
Density [kg/m^3^]	1.225
Viscosity [kg/ms]	1.7894 × 10^−5^
Solution method	Pressure–velocity coupled
Gradient	Least squares-based
Pressure	Standard
Momentum	Second-order upwind
Turbulent Kinetic Energy—k	First-order upwind
Specific Dissipation Rate—ω	First-order upwind

**Table 7:** Attributes of the computational mesh of the flow domain and boundary layer in the case of the feathered blade ($\vec{V_{\infty }}$ = 35 m/s).

Mesh Attribute	Value
Number of elements	280,202
Surface size [m]	0.3
Inflation—first layer height [m]	0.3
Number of layers of prismatic elements (inflation—layers)	20
Orthogonal quality	0.95
Minimum orthogonal quality	0.383
Skewness average	0.0098
Maximum skewness	0.53

**Table 8:** Attributes of the computational mesh of the flow domain and boundary layer in the case of operation in the rated conditions ($\vec{V_{\infty }}$ = 10.59 m/s).

	Coarse Mesh	Fine Mesh
Mesh Attribute	Value	
Number of elements	307,741	3,106,827
Number of nodes	1,634,325	16,684,340
Surface element size [m]	0.6	0.3
Body of influence size [m]	1.5	0.8
Number of inflation layers	10	20
Orthogonal quality	0.956	0.966
Minimum rectangular quality (Minimum orthogonal quality)	0.150	0.237
Skewness	0.0200	0.0115
Maximum skewness	0.900	0.867
$\mathrm{Thrust}\;[\mathrm{MN}]\;\vec{T}$	0.66	0.73
$\mathrm{Flapwise}\;\mathrm{bending}\;\mathrm{moment}\;[\mathrm{MNm}]\;{\vec{M}}_{flap}$	−52.0	−58.7

**Table 9:** Linear interpolation for the thrust [11].

$\mathrm{Wind}\;\mathrm{Velocity}\;\vec{V_{\infty }}$ [m/s]	$\mathrm{Thrust}\;\mathrm{of}\;\mathrm{Rotor}\;{\vec{T}}_{rotor}$ [MN]
10.2096	2.2644
10.6584	2.4473

**Table 10:** Linear interpolation for the flapwise bending moment [11].

$\mathrm{Wind}\;\mathrm{Velocity}\;\vec{V_{\infty }}$ [m/s]	$\mathrm{Flapwise}\;\mathrm{Bending}\;\mathrm{Moment}\;{\vec{M}}_{flap}$ [MNm]
10.2096	60.3222
10.6584	65.1486

**Table 11:** Structural analysis mesh properties.

Attribute	Value
Number of elements	40,156
Nodes	122,929
Area size [m]	0.2
Average skewness	0.06
Element quality	0.947

**Table 12:** Maximum tensile strength for materials [42,43,44].

Material/Fiber	Ultimate Tensile Strength (UTS) [MPa]
Carbon Fiber	1800–2400
E-Glass	500–800
S-2-Glass	1200–1500
Basalt	700–1200
Bamboo	350–500
Aramid	2000–2400

**Table 13:** Maximum equivalent von Mises stresses and maximum total displacement for each blade skin scenario.

Blade Skin Material	E-Glass		S-2-Glass		Aramid		Basalt		Bamboo	
$\mathrm{Wind}\;\mathrm{Velocity}\;\vec{V_{\infty }}$ [m/s]	10.59	35	10.59	35	10.59	35	10.59	35	10.59	35
Equivalent von Mises stresses (maximum) [MPa]	93.76	12.63	94.01	12.67	95.88	12.65	94.42	12.7	133	17.94
Maximum total displacement [m]	2.467	0.2374	2.098	0.2035	1.333	0.1571	2.190	0.2053	4.834	0.8078
Maximum shear stress [MPa]	45.29	7.00	45.43	7.02	46.26	7.06	45.44	7.04	45.44	9.62

## Data Availability

The original contributions presented in this study are included in the article. Further inquiries can be directed to the corresponding author.
